# Association between family environment and emergence delirium in pediatric patients after tonsillectomy and adenoidectomy: an observational prospective study

**DOI:** 10.1016/j.bjane.2025.844676

**Published:** 2025-09-03

**Authors:** Yubo Gao, Huihui Pei, Zhendong Liu, Yunfeng Bai, Jun Liu, Xinli Ni

**Affiliations:** General Hospital of Ningxia Medical University, Department of Anesthesiology and Perioperative Medicine, Xingqing District, China

**Keywords:** Child, Emergence delirium, Family characteristics, Risk factors

## Abstract

**Background:**

Preoperative anxiety in children is a known risk factor for Emergence Delirium (ED). The family environment may indirectly influence ED by modulating anxiety levels, but its direct role in ED remains unclear. The purpose of this study is to explore the associations between the occurrence of ED and family environmental factors in children. Identifying such associations may support the use of preoperative screening and targeted interventions to reduce ED risk.

**Methods:**

In this prospective observational study, 334 children (3∼7 years) undergoing elective tonsillectomy/adenoidectomy were assessed. Preoperative visits recorded clinical data and used the Chinese Family Environment Scale (FES-CV) and State/Trait Anxiety Inventories (parental anxiety). Preoperative child anxiety was measured via modified Yale Preoperative Anxiety Scale (m-YPAS). ED was assessed postoperatively in PACU using the Pediatric Anesthesia Emergence Delirium scale (PAED ≥ 10).

**Results:**

ED incidence was 21.9%. No significant association existed between overall home environment and ED. However, achievement orientation (FES-CV) negatively correlated with the m-YPAS score (m-YPAS; *r* = -0.139, p = 0.011). Independent ED risk factors identified: younger age (OR = 0.949, 95% CI 0.912∼0.988), introverted personality (OR = 0.393, 95% CI 0.184∼0.843), and higher postoperative pain (FLACC score; OR = 1.885, 95% CI 1.610∼2.208).

**Conclusion:**

While no direct link between home environment and ED was found, the negative correlation between achievement orientation and preoperative anxiety suggests an indirect influence. Identifying high-risk children using factors like age, personality, and pain levels remains important for ED prevention.

## Introduction

Tonsillectomy and Adenoidectomy (T&A) is one of the most common surgeries in otolaryngology and is widely performed to treat chronic tonsillitis and obstructive sleep apnea. The procedure is especially effective in preschool-aged children.^1^ However, children often experience Emergence Delirium (ED) after T&A, which is an early behavioral change following general anesthesia, primarily characterized by perceptual disturbances and psychomotor agitation. This phenomenon is particularly common in preschool-aged children.^2^ Previous studies have shown that the incidence of ED in pediatric patients following anesthesia ranges from 1.3% to 84.4%.^3^, ^4^, ^5^ The large variation in incidence reflects the individual variability in the occurrence of ED. Still, the consequences of ED should not be underestimated, as the condition may lead to self-harm, surgical site rupture, and displacement of indwelling catheters. Consequently, hospital stay may be extended, increasing medical costs and potentially having long-term effects on the psychological and physiological health of children.^6^

The occurrence of ED is influenced by various factors, including the child's age, temperament, preoperative anxiety levels, type of surgery, and postoperative pain.^7^ Recent studies have reported that children with high preoperative anxiety have a higher incidence of postoperative ED and experience a more painful, slower, and more complex recovery process.^7^^,^^8^ The family environment, as the child's earliest living environment, has a profound impact on psychological and social development. Factors such as emotional support provided by the family, lifestyle, and the quality of postoperative care may all be closely related to the occurrence and development of ED.^9^^,^^10^ The impact of the family environment on children's mental health is widely recognized. Nonetheless, no study to date has explored the effects of family environmental factors on the incidence of ED or whether these factors affect the occurrence of ED by influencing preoperative anxiety levels.

Therefore, this study aims to explore the correlation between family environmental factors and the occurrence of postoperative ED in preschool children undergoing T&A, as well as to investigate other potential risk factors affecting the occurrence of ED.

## Methods

### Study design

**Figure 1:**
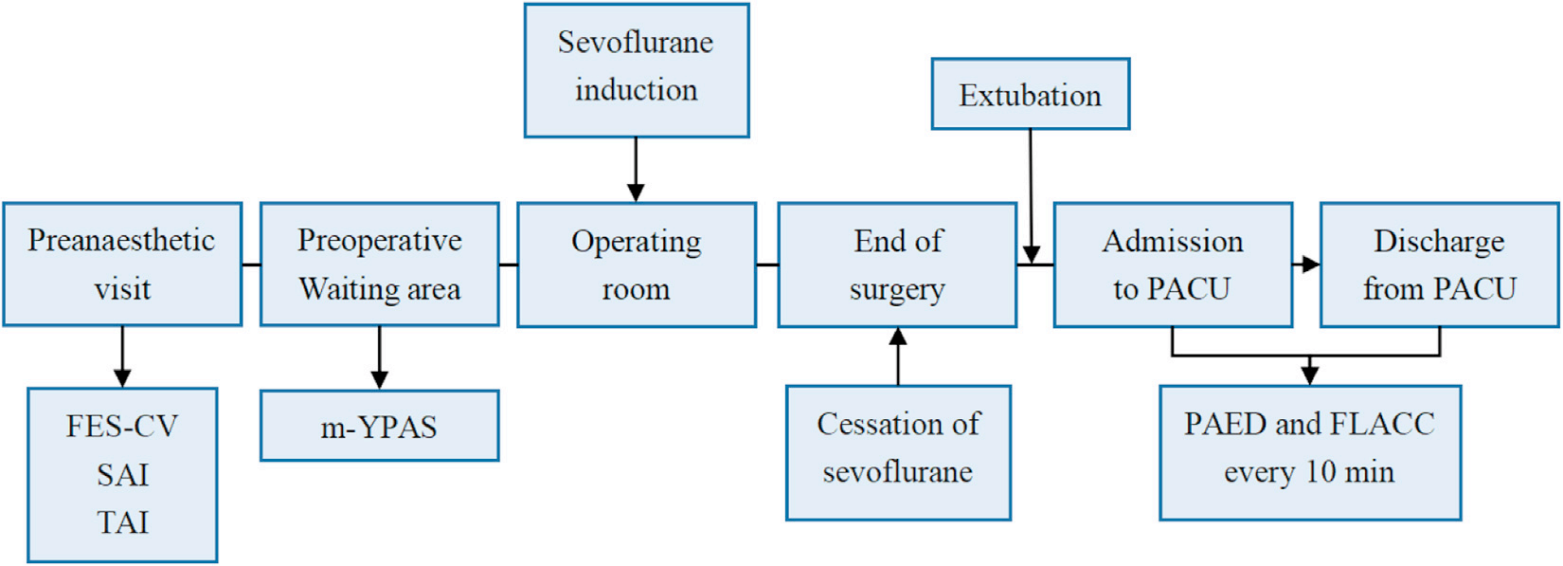
Flowchart of assessment according to the timeline. FES-CV, Family Environment Scale-Chinese Version; SAI, State Anxiety Inventory; TAI, Trait Anxiety Inventory; m-YPAS, modified Yale Preoperative Anxiety Scale; PACU, Post-anesthesia Care Unit; PAED, Pediatric Emergence Delirium; FLACC, Face, Legs, Activity, Crying, Consolability.

This prospective, single-center observational study was conducted in accordance with the principles outlined in the Declaration of Helsinki. This study was approved by the Medical Research Ethics Review Committee of the General Hospital of Ningxia Medical University (KYLL-2024-0008) (http://www.nyfy.com.cn/) and registered at the Chinese Clinical Trial Registry on July 15, 2024 (ChiCTR2400086958) (http://www.chictr.org.cn). The study period spanned from January 2024 to September 2024. One day before surgery, trained researchers conducted preoperative visits, explaining the purpose, methods, and confidentiality principles of the study to the parents. The anesthesia process and risks were explained, and informed consent was obtained. Moreover, the children's general clinical data were recorded; the Family Environment Scale-Chinese Version (FES-CV) was used to assess the family environment, and the State Anxiety Inventory (SAI) and Trait Anxiety Inventory (TAI) were employed to investigate the parents' anxiety levels.^11^ The occurrence of ED in children was evaluated using the Pediatric Anesthesia Emergence Delirium (PAED) scale after surgery.^12^ The correlation between family environmental factors, other clinical data, and ED was analyzed (Fig. 1).

### Sample size

Based on the literature review and clinical observations, this study intends to examine approximately four variables to analyze risk factors for ED. In unconditional logistic regression with a binary outcome, the sample size for the less frequent dependent variable should be at least 5∼10 times the number of variables included. Furthermore, prior research indicated that the incidence of ED in pediatric patients undergoing T&A ranges from 13% to 28%.^13^^,^^14^ This study adopted the higher estimate of 13%. Consequently, the minimum sample size required was approximately 308, calculated as 4 × 10 ÷ 0.13. Accounting for a 5% attrition rate, the final sample size was determined to be 334 patients.

### Study population and inclusion, and exclusion criteria

Inclusion criteria for this study: 1) Age 3∼7 years; 2) American Society of Anesthesiologists (ASA) classification I∼II; 3) Scheduled for elective T&A under general anesthesia, performed by the same surgeon; 4) Expected surgical duration not exceeding 2 hours; 5) Parents of the child could communicate normally and showed normal cognitive abilities, and independently completed the relevant questionnaires; 6) Parents of the child agreed to participate in this study and provided signed informed consent form. Exclusion criteria: 1) Refusal to participate in the study; 2) Congenital or other genetic diseases affecting brain development; 3) Either parent of the child had psychological issues such as mental abnormalities; 4) Either parent was not able to complete the relevant questionnaire due to other reasons; 5) Unanticipated cessation of surgery; 6) Incomplete data affecting judgment; 7) Observers actively withdrew from the study.

### Anesthesia

All children fasted for 6 hours and avoided clear liquids for 2 hours. In the preoperative waiting area, the researchers assessed the children's preoperative anxiety levels using the Modified Yale Preoperative Anxiety Scale (m-YPAS).^15^ Anesthesia was managed by a designated anesthetist according to a unified inhalation and intravenous anesthesia plan. Upon arrival in the operating room, standard monitoring was initiated, including non-invasive blood pressure, pulse oximetry, electrocardiogram, and heart rate. Subsequently, 8% sevoflurane was administered with an oxygen flow rate of 6 L/min. Once the child lost consciousness and spontaneous movement ceased, a peripheral venous access was established. Midazolam 0.05 mg.kg^-1^, sufentanil 0.03 µg.kg^-1^, etomidate 0.5 mg.kg^-1^, propofol 2 mg.kg^-1^, and rocuronium 0.6 mg.kg^-1^ were administered intravenously, and an appropriately sized endotracheal tube was inserted. Propofol was continuously infused at 4∼6 mg.kg^-1^.h^-1^ and remifentanil at 0.1∼0.2 µg.kg^-1^.min^-1^, while 0.3%∼0.5% sevoflurane was continuously inhaled. Heart rate or blood pressure fluctuations of more than 20% above baseline were corrected by adjustments to the infusion rates of propofol and remifentanil and to the inhalation concentration of sevoflurane. During the surgery, intermittent doses of rocuronium 0.15 mg.kg^-1^ were administered according to the metabolism time of rocuronium and the needs of the procedure. The anesthesia machine was set to volume control ventilation, with an airway pressure of 10∼20 cmH_2_O, tidal volume of 6∼10 mL.kg^-1^, and a respiratory rate of 20∼25 breaths.min^-1^. The tidal volume and respiratory rate were adjusted to maintain PETCO_2_ at 35∼45 mmHg. At the end of the surgery, the infusions of propofol, remifentanil, and inhaled sevoflurane were stopped. Oral secretions were fully suctioned, and sugammadex 2∼4 mg^-1^ and flumazenil 0.01 mg^-1^ were administered. After stabilization of hemodynamics and restoration of consciousness, the endotracheal tube was removed, and vital signs were closely monitored. After confirming that the child's vital signs were stable, the patient was transferred to the Post-Anesthesia Care Unit (PACU) for further observation (Fig. 1).

### Predictive assessment tool

The Family Environment Scale (FES),^16^ developed by American psychologists Moss et al., was revised and adapted by LP Fei et al. in 1991 into its Chinese version. The assessment of the family environment utilizes the FES-CV, which comprises ten dimensions: cohesion, expressiveness, conflict, independence, achievement orientation, intellectual-cultural orientation, active-recreational orientation, moral-religious emphasis, organization, and control, each dimension containing nine items.

### Study outcomes

The primary outcome is the correlation between family environmental factors and ED. The secondary outcomes include the incidence of ED, the general condition of children before surgery, anesthesia, operation time, and the correlation between postoperative pain and ED.

Flowchart of assessment according to the timeline is shown in Figure 1.

### Statistical analysis

Software SPSS 26.0 (SPSS Inc., Armonk, NY, USA) and GraphPad Prism 9.0 (GraphPad Inc., California, USA) were used for data analysis. The normality of quantitative data was evaluated using the Kolmogorov-Smirnov (K-S) test, with p ≥ 0.05 indicating normal distribution. Quantitative variables conforming to a normal distribution were presented as mean ± Standard Deviation (SD), and comparisons between groups were conducted using the independent samples *t*-test. Non-normally distributed quantitative data were presented as medians with Interquartile Ranges (IQR), and comparisons between groups were conducted using the Mann-Whitney *U* test. Categorical data were presented as the number of cases (%) and compared using the Chi-Square test or Fisher’s exact test. Univariate and multivariate logistic regression analyses were performed to identify the risk factors affecting the occurrence of ED. Variables showing p < 0.05 in the univariate analysis were incorporated into the multivariate binary logistic regression equation for further analysis. Additionally, *R* language was used to create forest plots and correlation heatmaps for data visualization.

## Results

**Figure 2:**
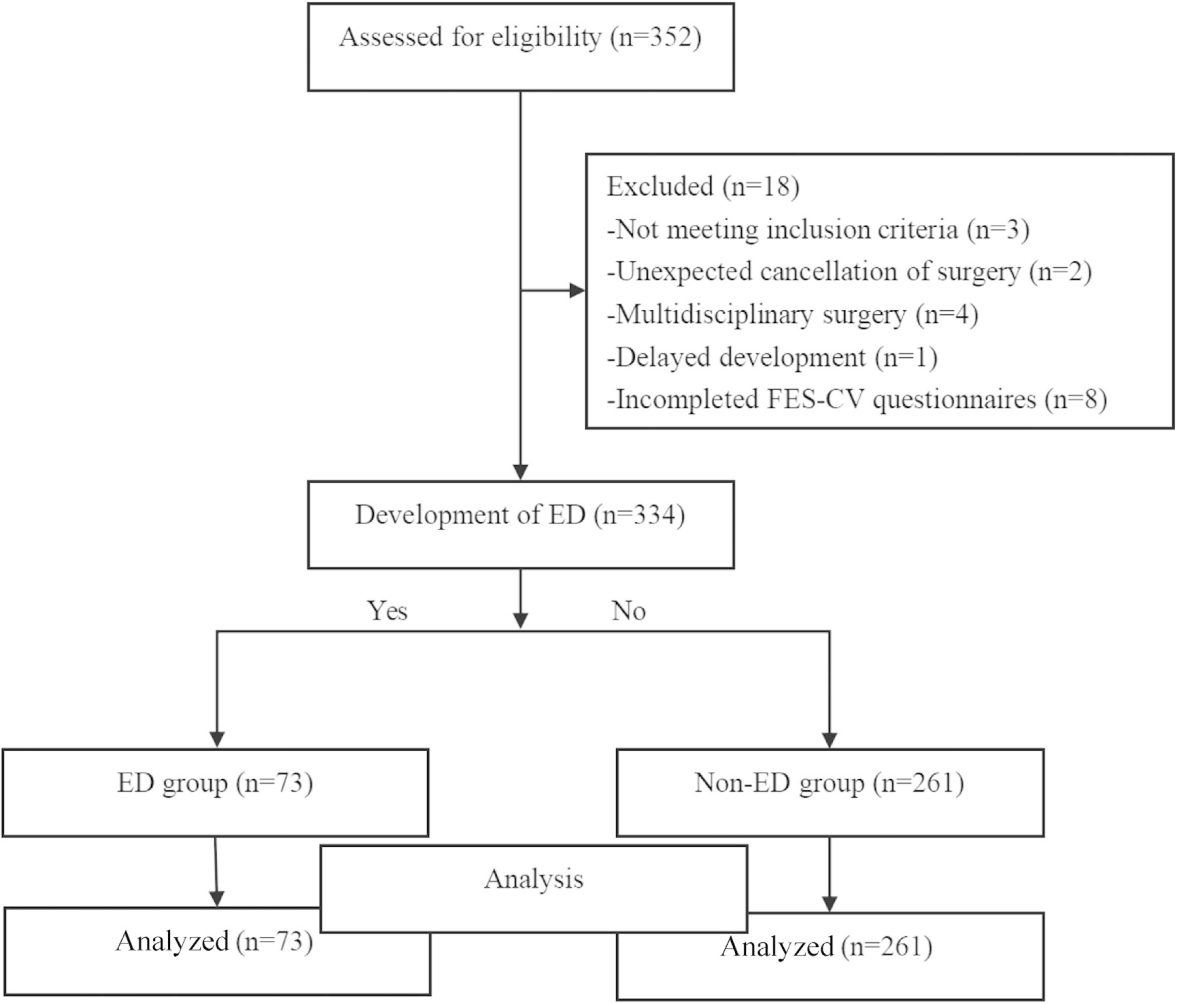
Study flow diagram. Patients involved in the study and the respective groups analyzed. ED, Emergence Delirium; FES-CV, Family Environment Scale-Chinese Version.

In this study, a total of 352 children were initially selected. Figure 2 displays the study flow diagram. Among them, four were excluded for not meeting the inclusion criteria; one was excluded due to developmental delay; five were excluded as their parents failed to complete the relevant questionnaires; and eight were excluded due to unplanned surgical termination. After these screenings, a total of 334 children were included for analysis. Ultimately, 73 cases of ED were detected, corresponding to an incidence rate of 21.9% (Fig. 2).

### Patient demographics

Table 1 shows the perioperative demographic and clinical data of the patient population.

**Table 1:** Demographic and clinical characteristics of study participants.

	ED group (n = 73)	Non-ED group (n = 261)	χ^2^/*t*	p-value
**Sex, n (%)**			0.157	0.692
Male	46 (63.01)	171 (65.52)		
Female	27 (36.99)	90 (34.48)		
**Age (mouth), mean ± SD**	61.52 ± 14.56	71.78 ± 15.03	5.189	< 0.001^b^
**BMI (kg.m^-2^), mean ± SD**	16.15 ± 3.15	15.80 ± 2.54	-0.980	0.328
**ASA physical status, n (%)**			0.018	0.893
Ⅰ	52 (71.23)	188 (72.03)		
Ⅱ	21 (28.77)	73 (27.97)		
**History of surgery, n (%)**			0.043	0.834
Yes	4 (5.48)	16 (6.13)		
No	69 (94.52)	245 (93.87)		
**Only child status, n (%)**			0.059	0.808
Yes	26 (35.62)	97 (37.16)		
No	47 (64.38)	164 (62.84)		
**Single-parent child, n (%)**			0.405	0.535
Yes	6 (8.22)	16 (6.13)		
No	67 (91.78)	245 (93.87)		
**Children personality, n (%)**			5.219	0.022^a^
Introversion	30 (41.10)	71 (27.20)		
Extroversion	43 (58.90)	190 (72.80)		
**Educational experience, n (%)**			10.714	0.003^a^
No educational experience	6 (8.22)	12 (4.60)		
Kindergarten	58 (79.45)	168 (64.37)		
Primary school	9 (12.33)	81 (31.03)		
**Mother’s education level, n (%)**			0.546	0.460
Below college level	36 (49.32)	116 (44.44)		
College diploma or above	37 (50.68)	145 (55.56)		
**Father’s education level, n (%)**			0.658	0.417
Below college level	33 (45.21)	132 (50.57)		
College diploma or above	40 (54.79)	129 (49.43)		
**Residence, n (%)**			0.006	0.941
County town	30 (41.10)	106 (40.61)		
City	43 (58.90)	155 (59.39)		
**Anesthesia time (min), mean ± SD**	65.68 ± 16.00	60.30 ± 18.29	-2.285	0.023^a^
**Surgical time (min), mean ± SD**	47.79 ± 16.02	41.67 ± 17.12	-2.740	0.006^a^
**Total hospitalization duration (day), mean ± SD**	2.88 ± 1.68	2.87 ± 1.79	-0.013	0.989
**Postoperative hospitalization duration (day), mean ± SD**	1.55 ± 0.88	1.72 ± 1.01	1.325	0.186
**SAI score, mean ± SD**	43.40 ± 8.93	43.51 ± 9.19	0.090	0.929
**TAI score, mean ± SD**	43.60 ± 8.69	43.51 ± 8.77	-0.077	0.939
**m-YPAS score, mean ± SD**	41.29 ± 19.50	35.45 ± 16.13	-2.346	0.021^a^
**FLACC score, mean ± SD**	5.01 ± 3.08	1.13 ± 1.49	-10.447	< 0.001^b^

ED, Emergence Delirium; ASA, American Society of Anesthesiologists; BMI, Body Mass Index; m-YPAS, Modified Yale Preoperative Anxiety Scale; FLACC, Face, Legs, Activity, Cry, Consolability; SAI, State Anxiety Inventory; TAI, Trait Anxiety Inventory.

a p < 0.05.

b p < 0.001.

Compared to the non-ED group, the children in the ED group were significantly younger and had a lower level of schooling (p < 0.05). Children with introverted personalities were more likely to experience ED (p = 0.022), with higher m-YPAS and FLACC scores (p < 0.05) and longer anesthesia and surgery times (p < 0.05) (Table 1).

### Primary and secondary outcomes

Table 2 summarizes the key outcomes. No significant differences were observed in the dimensions of FES-CV between the ED group and the non-ED group in terms of cohesion, expressiveness, conflict, independence, achievement orientation, intellectual-cultural orientation, active-recreational orientation, moral-religious emphasis, organization, and control (p > 0.05). Nonetheless, the measures of cohesion, achievement orientation, intellectual-cultural orientation, and control in the ED group were slightly lower than those in the non-ED group. Conversely, the levels of expressiveness, conflict, independence, active-recreational orientation, moral-religious emphasis, and organization showed a higher trend in the ED group compared to the non-ED group (Table 2).

**Table 2:** Family environmental factors associated with ED.

	ED group (n = 73)	Non-ED group (n = 261)	*t*	p-value
**Cohesion (score), mean ± SD**	8.11 ± 1.24	8.15 ± 1.37	0.224	0.823
**Expressiveness (score), mean ± SD**	5.86 ± 1.34	5.62 ± 1.38	-1.312	0.190
**Conflict (score), mean ± SD**	2.66 ± 1.62	2.46 ± 1.69	-0.910	0.363
**Independence (score), mean ± SD**	5.75 ± 1.40	5.65 ± 1.36	-0.565	0.573
**Achievement orientation (score), mean ± SD**	6.18 ± 1.23	6.29 ± 1.78	0.624	0.534
**Intellectual-cultural orientation (score), mean ± SD**	4.77 ± 1.93	4.96 ± 1.91	0.768	0.443
**Active-recreational orientation (score), mean ± SD**	6.45 ± 1.86	6.38 ± 2.10	-0.283	0.778
**Moral-religious emphasis (score), mean ± SD**	5.74 ± 1.34	5.61 ± 1.42	-0.683	0.495
**Organization (score), mean ± SD**	6.90 ± 1.40	6.83 ± 1.69	-0.375	0.708
**Control (score), mean ± SD**	4.32 ± 2.05	4.43 ± 1.92	0.428	0.669

ED, Emergence Delirium.

**Figure 3:**
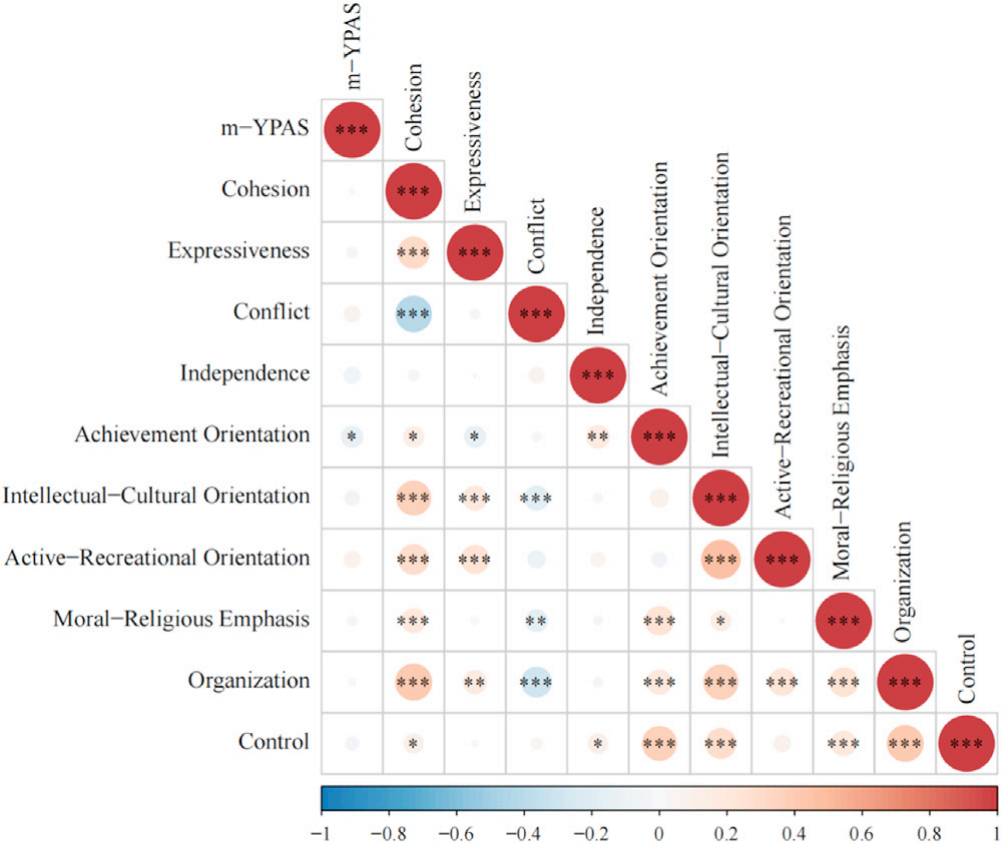
Association of family environmental factors with preoperative anxiety in children. m-YPAS, modified Yale Preoperative Anxiety Scale.

Table 3 displays the correlation between each dimension of the FES-CV scale and the m-YPAS scores of the children. This study revealed that the achievement orientation scores in the family environment scale were significantly negatively correlated with the m-YPAS scores (*r* = -0.139, p = 0.011). A correlation heatmap was generated using the corrplot package in *R*, with red indicating a positive correlation, blue indicating a negative correlation, and darker colors representing stronger correlations (Fig. 3).

**Table 3:** The correlation between the dimensions of the FES-CV scale and the children's m-YPAS scores.

	m-YPAS	
	r	p-value
**m-YPAS**	1	
**Cohesion**	0.009	0.870
**Expressiveness**	0.033	0.551
**Conflict**	0.077	0.158
**Independence**	-0.078	0.156
**Achievement orientation**	-0.139	0.011^a^
**Intellectual-cultural Orientation**	-0.062	0.261
**Active-recreational Orientation**	0.076	0.164
**Moral-religious emphasis**	-0.025	0.647
**Organization**	-0.015	0.784
**Control**	-0.050	0.364

m-YPAS, Modified Yale Preoperative Anxiety Scale.

a p < 0.05, ^b^ p < 0.001.

**Figure 4:**
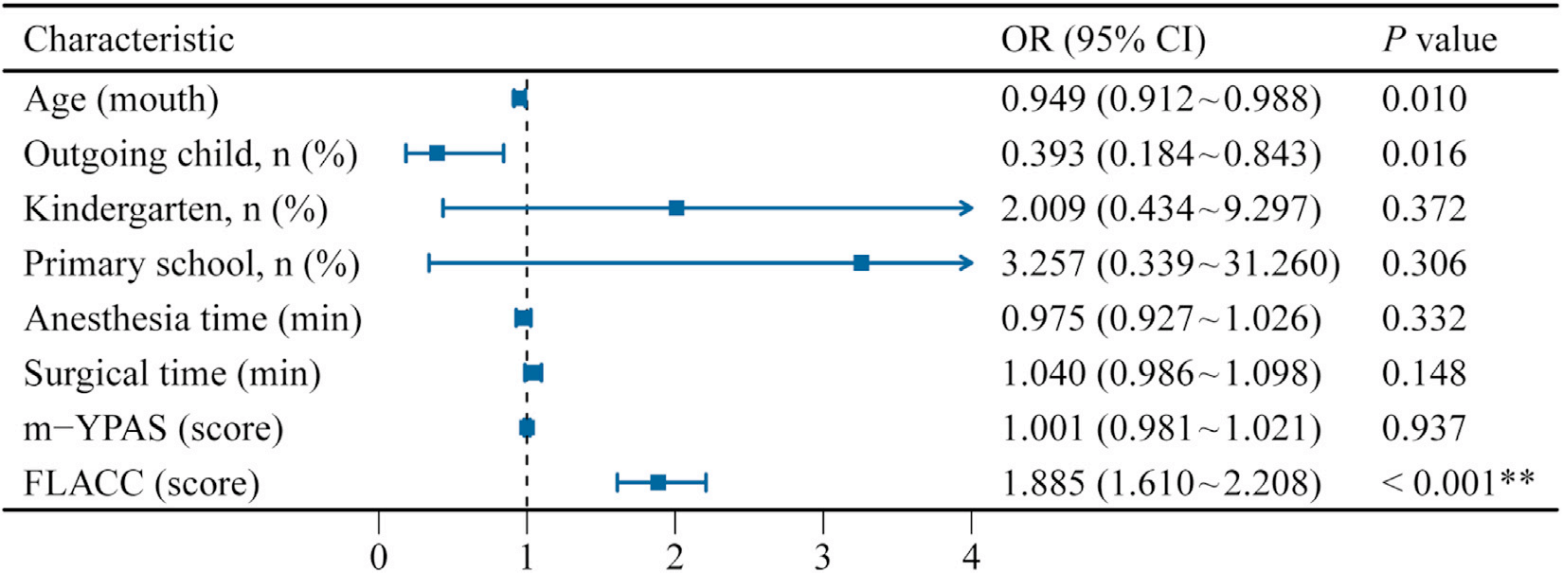
Forest plot of the binary variable predicting the occurrence of ED. ED, emergence delirium; m-YPAS, Modified Yale Preoperative Anxiety Scale; FLACC, Face, Legs, Activity, Cry, Consolability; CI, Confidence Interval; OR, Odds Ratio. * p < 0.05, ** p < 0.001.

Table 4 presents the multifactor binary logistic regression analysis predicting ED. Indicators with statistically significant differences between the two groups were included in the binary logistic regression model for analysis. The occurrence of ED post-T&A in preschool children was set as the dependent variable and was assigned a value of 1 for occurrence or 0 for non-occurrence. A multifactor binary logistic regression analysis was performed to identify the risk factors. The results indicated that younger age, higher FLACC scores, and an introverted personality in children were independent risk factors for the occurrence of ED after T&A in preschool children (p < 0.05). A forest plot was drawn using the forestplotor in *R* language, revealing that child age (OR = 0.949, 95% CI 0.912∼0.988), personality (OR = 0.393, 95% CI 0.184∼0.843), and FLACC score (OR = 1.885, 95% CI 1.610∼2.208) were independent risk factors for the occurrence of ED after T&A (Fig. 4).

**Table 4:** Multifactor binary logistic regression analysis predicting ED.

	β	SE	Wald χ^2^	p-value	OR value	95% CI
**Age (mouth)**	-0.052	0.020	6.591	0.010^a^	0.949	0.912∼0.988
**Outgoing child, n (%)**	-0.933	0.389	5.760	0.016^a^	0.393	0.184∼0.843
**Kindergarten, n (%)**	0.698	0.782	0.796	0.372	2.009	0.434∼9.297
**Primary school, n (%)**	1.181	1.154	1.047	0.306	3.257	0.339∼31.260
**Anesthesia time (min)**	0.000	0.026	0.941	0.332	0.975	0.927∼1.026
**Surgical time (min)**	0.040	0.027	2.095	0.148	1.040	0.986∼1.098
**m-YPAS (score)**	0.001	0.010	0.006	0.937	1.001	0.981∼1.021
**FLACC (score)**	0.634	0.081	62.020	< 0.001^b^	1.885	1.610∼2.208

ED, Emergence Delirium; m-YPAS, Modified Yale Preoperative Anxiety Scale; FLACC, Face, Legs, Activity, Cry, Consolability; CI, Confidence Interval; OR, Odds Ratio.

a p < 0.05,

b p < 0.001.

## Discussion

The incidence of ED in children aged 3 to 7 years undergoing T&A in this study was 21.9%. The results revealed that younger age, introverted personality, and postoperative pain are independent risk factors for ED in children undergoing T&A. However, this study found no direct evidence of an association between family environment and ED.

The family environment is the first environment that children come into contact with, playing a decisive role in their growth and development.^9^^,^^10^ In terms of psychological development, a warm and loving family gives children a sense of security and belonging, which promotes positive emotions and good character in children.^17^ In contrast, a poor family environment may lead to negative emotions, such as anxiety, fear, and inferiority.^18^ Research has shown that anxiety in children is largely influenced by environmental factors.^19^ Furthermore, studies have found that the family environment is an early risk factor for emotional disorders in children.^20^^,^^21^

This study employed the FES-CV scale to evaluate family environment. Notably, the dimension of achievement orientation was significantly negatively correlated with preoperative anxiety in children. Families with higher achievement orientation tend to emphasize effort and progress, set positive examples for their children, and provide stable psychological support.^22^ This family environment may help reduce preoperative anxiety levels in children, thereby playing a preventive role in the occurrence of ED. Children with preoperative anxiety are more susceptible to stress responses both physiologically and psychologically, which increases the risk of postoperative ED.^7^ For every 10-point increase in the children's m-YPAS score, the risk of developing ED increases by 10%.^15^ Interactive videos can alleviate children's preoperative anxiety, thereby reducing the incidence of ED.^15^ Therefore, families with a higher achievement orientation environment may be indirectly associated with a lower incidence of postoperative ED by lowering children's preoperative anxiety.

Although this study did not find a direct association between the various dimensions of the family environment scale and post-operative ED, a close connection was observed between achievement orientation and pre-operative anxiety. This finding provides a new perspective on the relationship between the family environment and children's post-operative recovery and offers potential directions for clinical intervention. Future research could further explore how improving the achievement orientation of the family environment can reduce pre-operative anxiety in children, providing a potential method for reducing the occurrence of post-operative ED.

Age is considered an important factor influencing the occurrence of ED.^23^ This may be due to the incomplete brain development in younger children, who are more sensitive to external stimuli. During the brain development process, the hippocampus and cholinergic system are key neural structures that play important roles in cognitive and behavioral regulation. A reduction in the number of neurons in the locus coeruleus and gray matter can lead to decreased levels of neurotransmitters such as norepinephrine, acetylcholine, dopamine, and gamma-aminobutyric acid. This reduction in neurotransmitters may affect the cognitive function of children, leading to a higher incidence of ED.^24^ Low effort control in children may be associated with ED (OR = 2.12, 95% CI 0.88∼5.10).^25^ The unique temperament of children influences their responses to stimuli, which is a result of the interaction between children and their environment. The results of this study reveal that children with an extroverted personality are better communicators and have a lower incidence of ED. Postoperative pain is one of the main risk factors for ED in children.^26^ Pain can cause changes in brain wave patterns, specifically an increase in δ and γ wave activity, which in turn leads to the occurrence of ED.^27^ Once pain is effectively controlled, the incidence of ED in children can be significantly reduced.^26^

### Limitations

Firstly, the family environment factors included are not comprehensive enough, which may affect the accuracy of the results. Secondly, the FES-CV scale includes many items, which may lead to respondent fatigue, leading to random answers and affecting the accuracy of the family environment assessment. Finally, this research is a single-center study that only includes children undergoing T&A. Therefore, larger-scale, multi-center studies are needed to further validate and expand these findings.

## Conclusions

This study found that the achievement orientation rate in the FES-CV scale is significantly negatively correlated with the preoperative anxiety level of children, while introverted personality, younger age, and higher postoperative FLACC scores are independent risk factors for the occurrence of ED. Although no direct association was found between family environment and ED, achievement orientation may indirectly affect ED by influencing the psychological state of children. Therefore, optimizing the family environment and conducting preoperative screening and psychological intervention for high-risk children may help reduce the incidence of ED.

## Data availability

All data analyzed during this study are available from the corresponding author on reasonable request.

## Authors’ contributions

XN contributed to the conception and design of the review. YG and HP contributed the case collection and manuscript writing. ZL and YB contributed equally to the overall text and figures. JL helped the statistical analysis of data. All authors contributed to the article and approved the final version.

## Funding

This work was funded in part by grants from the Natural Science Foundation of Ningxia Hui Autonomous Region, China (Grant n° 2024AAC03547).

## Conflicts of interest

The authors declare no conflicts of interest.

## Appendix Supplementary materials

Image, application 1
