# Supplementary material for: Association between family environment and emergence delirium in pediatric patients after tonsillectomy and adenoidectomy: an observational prospective study
Source: Braz J Anesthesiol. 2025 Sep 3;75(6):844676. doi: 10.1016/j.bjane.2025.844676 (PMC12508891; doi:10.1016/j.bjane.2025.844676)
Supplement: Supplementary file 1 [file mmc1.docx]

**BJAN-D-25-00136**

**Supplementary Material**

**Family Environment Scale-Chinese Version**

Instructions: This questionnaire is designed to understand your views on your family. Please determine whether the following questions correspond to the actual situation in your home. If you believe a question matches the actual situation of your family, please answer “yes”. If it does not match or basically does not match, please answer “no”. If it is difficult to judge whether it matches, you should answer based on the behavior of the majority of family members or the situation that frequently occurs. If you still cannot determine, please answer according to your own estimate. Please be sure to answer every question. Some questions are marked with an “*”, indicating that the sentence has a negative meaning. Please pay attention to correctly understanding the content of the sentence. Remember, the “family” referred to in this questionnaire means the small family that you live and eat with. When answering the questionnaire, do not speculate on others' views of your family; please answer according to the actual situation.

| **Family Environment Scale-Chinese Version** | | | |
| --- | --- | --- | --- |
| **Item** | | **Yes** | **No** |
| 1 | We always give the greatest help and support to each other in our family. | Yes□ | No□ |
| 2 | Family members always keep their feelings to themselves and do not reveal them to other family members. | Yes□ | No□ |
| 3 | There are frequent quarrels in our home. | Yes□ | No□ |
| *4 | We rarely engage in individual activities at home. | Yes□ | No□ |
| 5 | Family members always do their best in whatever they do. | Yes□ | No□ |
| 6 | We often discuss political and social issues in our family. | Yes□ | No□ |
| 7 | Most weekends and evenings, family members spend time at home instead of going out for social and entertainment activities. | Yes□ | No□ |
| 8 | We all believe that no matter how difficult it is, children should first meet the various needs of the elderly. | Yes□ | No□ |
| 9 | Major activities in the family are carefully planned. | Yes□ | No□ |
| *10 | Family members rarely insist that others follow the family rules. | Yes□ | No□ |
| 11 | We feel bored at home. | Yes□ | No□ |
| 12 | We can say whatever we want at home. | Yes□ | No□ |
| *13 | Family members rarely show anger openly to each other. | Yes□ | No□ |
| 14 | We strongly encourage family members to be independent. | Yes□ | No□ |
| 15 | In order to have a good future, family members devote almost all their energy. | Yes□ | No□ |
| *16 | We rarely go out to attend lectures, watch movies, or visit museums and exhibitions. | Yes□ | No□ |
| 17 | Family members often go to friends' homes to play and have meals together. | Yes□ | No□ |
| 18 | Family members all believe that actions should conform to social trends. | Yes□ | No□ |
| 19 | Generally speaking, we all pay attention to keeping the house tidy. | Yes□ | No□ |
| *20 | There are few fixed routines and family rules in our home. | Yes□ | No□ |
| 21 | Family members are willing to devote a lot of energy to household matters. | Yes□ | No□ |
| 22 | Complaining at home easily makes family members annoyed. | Yes□ | No□ |
| 23 | Sometimes family members break things when they are angry. | Yes□ | No□ |
| 24 | Family members all think independently. | Yes□ | No□ |
| 25 | Family members all believe that improving the standard of living is more important than anything else. | Yes□ | No□ |
| 26 | We all believe that learning new knowledge is more important than anything else. | Yes□ | No□ |
| *27 | No one in the family participates in various sports activities. | Yes□ | No□ |
| 28 | Family members often help the elderly and disabled people around them in daily life. | Yes□ | No□ |
| 29 | In our family, it is often difficult to find things when we need them. | Yes□ | No□ |
| 30 | Mealtimes and bedtimes in our family are always the same. | Yes□ | No□ |
| 31 | There is a harmonious and consistent atmosphere in our family. | Yes□ | No□ |
| 32 | Everyone in the family can talk about their difficulties and worries. | Yes□ | No□ |
| *33 | Family members rarely lose their temper with each other. | Yes□ | No□ |
| 34 | Everyone in our family is completely free to come and go. | Yes□ | No□ |
| 35 | We all believe that competition is a good thing in any situation. | Yes□ | No□ |
| *36 | We are not very interested in cultural activities. | Yes□ | No□ |
| 37 | We often go to the movies, watch sports games, or go on outings. | Yes□ | No□ |
| 38 | We think that bribery is an acceptable phenomenon. | Yes□ | No□ |
| 39 | In our family, we place great emphasis on punctuality. | Yes□ | No□ |
| 40 | We have a fixed way of doing everything in our family. | Yes□ | No□ |
| *41 | Few people volunteer to do things when there is something to do at home. | Yes□ | No□ |
| 42 | Family members often openly express their feelings towards each other. | Yes□ | No□ |
| 43 | Family members often blame and criticize each other. | Yes□ | No□ |
| *44 | Family members rarely consider the opinions of other family members when doing things. | Yes□ | No□ |
| 45 | We are always constantly reflecting on ourselves and forcing ourselves to do better each time. | Yes□ | No□ |
| *46 | We rarely discuss issues related to scientific and technological knowledge. | Yes□ | No□ |
| 47 | Each member of our family is particularly interested in 1-2 types of entertainment activities. | Yes□ | No□ |
| 48 | We believe that no matter what, the younger generation should accept the advice of the older generation. | Yes□ | No□ |
| 49 | Members of our family often change their plans. | Yes□ | No□ |
| 50 | Our family places great emphasis on following fixed routines and family rules. | Yes□ | No□ |
| 51 | Family members always sincerely support each other. | Yes□ | No□ |
| 52 | If someone expresses dissatisfaction with household matters at home, someone will feel uncomfortable. | Yes□ | No□ |
| 53 | Family members sometimes fight with each other. | Yes□ | No□ |
| 54 | Family members rely on the help of other family members to solve the difficulties they encounter. | Yes□ | No□ |
| *55 | Family members are not very concerned about job promotions, academic performance, and other issues. | Yes□ | No□ |
| 56 | Someone in the family plays a musical instrument. | Yes□ | No□ |
| *57 | Family members do not often engage in entertainment activities other than work and study. | Yes□ | No□ |
| 58 | Family members voluntarily maintain public environmental hygiene. | Yes□ | No□ |
| 59 | Family members carefully keep their own rooms clean. | Yes□ | No□ |
| 60 | Family members can go out at night without consulting other family members in advance. | Yes□ | No□ |
| *61 | There is little collective spirit in our family. | Yes□ | No□ |
| 62 | We can openly discuss family financial issues in our family. | Yes□ | No□ |
| 63 | When family members have disagreements, we always avoid them to maintain harmony. | Yes□ | No□ |
| 64 | Family members hope that family members can solve problems independently. | Yes□ | No□ |
| *65 | Family members are not very enthusiastic about achieving success. | Yes□ | No□ |
| 66 | Family members often go to the library. | Yes□ | No□ |
| 67 | Family members sometimes participate in recreational learning according to personal hobbies or interests. | Yes□ | No□ |
| 68 | Family members all believe that they should strictly adhere to moral dogmas when handling affairs. | Yes□ | No□ |
| 69 | In our family, everyone's division of labor is clear. | Yes□ | No□ |
| *70 | There are no strict rules in our family to restrict us. | Yes□ | No□ |
| 71 | Family members always get along well with each other. | Yes□ | No□ |
| 72 | Family members are very careful when speaking to avoid hurting each other's feelings. | Yes□ | No□ |
| 73 | Family members often try to outdo each other. | Yes□ | No□ |
| 74 | If family members often engage in individual activities, it will hurt the feelings of other family members. | Yes□ | No□ |
| 75 | Work first, then enjoy is an old habit in our family. | Yes□ | No□ |
| 76 | Watching TV is more important than reading in our family. | Yes□ | No□ |
| 77 | Family members often participate in social activities outside the family in their spare time. | Yes□ | No□ |
| 78 | We believe that divorce is immoral no matter what. | Yes□ | No□ |
| *79 | Our family spends money without a plan. | Yes□ | No□ |
| 80 | The routines and family rules in our family cannot be changed. | Yes□ | No□ |
| 81 | Every member of the family always gets enough attention. | Yes□ | No□ |
| 82 | Our family often spontaneously discusses issues that are sensitive to family members. | Yes□ | No□ |
| 83 | When family members have conflicts, sometimes they argue loudly. | Yes□ | No□ |
| 84 | In our family, members are indeed encouraged to be free in their activities. | Yes□ | No□ |
| 85 | Family members often compare themselves with others to see who is better in study and work. | Yes□ | No□ |
| 86 | Family members are very fond of music, art, and literature. | Yes□ | No□ |
| 87 | Our way of entertainment is to watch TV and listen to the radio rather than go out. | Yes□ | No□ |
| 88 | We believe that improving the standard of living in the family is more important than strictly adhering to moral standards. | Yes□ | No□ |
| 89 | In our family, someone must wash the dishes immediately after meals. | Yes□ | No□ |
| 90 | Those who violate family rules will be severely criticized. | Yes□ | No□ |

Scoring and Analysis of the Scale: All 90 items are scored based on the selected answers. If the answer is “yes”, it is scored as “1” point. If the answer is “no”, it is scored as “2” points. The subscale scores are then calculated using the following method (“I~X” represents the score of item “X”).

Cohesion=(I~11-1)+(I~41-1)+(I~61-1)-[(1~1-2)+(I~21-2)+(I~31-2)+(I~51-2)+(I~71-2)+(I~81-2)]

Expressiveness = (I~2-1) + (I~22-1) + (I~52-1) + (I~72-1) - [(I~12-2) + (I~32-2) + (I~42-2) + (I~62-2) + (I~82-2)]

Conflict = (I~13-1) + (I~33-1) + (I~63-1) - [(I~3-2) + (I~23-2) + (I~43-2) + (I~53-2) + (I~73-2) + (I~83-2)]

Independence = (I~4-1) + (I~54-1) - [(I~14-2) + (I~24-2) + (I~34-2) + (I~44-2) + (I~64-2) + (I~74-2) + (I~84-2)]

Achievement Orientation = (I~55-1) + (I~65-1) - [(I~5-2) - (I~15-2) + (I~25-2) + (I~35-2) + (I~45-2) + (I~75-2) + (I~85-2)]

Intellectual-Cultural Orientation = (I~16-1) + (I~36-1) + (I~46-1) + (I~76-1) - [(I~6-2) + (I~26-2) + (I~56-2) + (I~66-2) + (I~86-2)]

Active-Recreational Orientation = (I~7-1) + (I~27-1) + (I~57-1) + (I~87-1) - [(I~17-2) + (I~37-2) + (I~47-2) + (I~67-2) + (I~77-2)]

Moral-Religious Emphasis = (I~18-1) + (I~38-1) + (I~88-1) - [(I~8-2) + (I~28-2) + (I~48-2) + (I~58-2) + (I~68-2) + (I~78-2)]

Organization = (I~29-1) + (I~49-1) + (I~79-1) - [(I~9-2) + (I~19-2) + (I~39-2) + (I~59-2) + (I~69-2) + (I~89-2)]

Control = (I~10-1) + (I~20-1) + (I~60-1) + (I~70-1) - [(I~30-2) + (I~40-2) + (I~50-2) + (I~80-2) + (I~90-2)]
